# Using Community Health Workers to Prevent Infectious Diseases in Women^1^

**DOI:** 10.3201/eid1011.040623_06

**Published:** 2004-11

**Authors:** Jamila Rashid, Olufemi O. Taiwo, Beatriz Barraza-Roppe, Maria Lemus

**Affiliations:** * Centers for Disease Control and Prevention, Atlanta, Georgia, USA;; † Somolu and Bariga Local Government, Lagos, Nigeria;; ‡ Instituto de Promotoras, San Diego, California, USA;; § Visión y Campromiso—The Community Health Worker/Promotoras Network, El Cerrito, California, USA

**Keywords:** Community Health Workers, Community Workers, Community healthcare workers, Promotoras, Outreach workers

Community health workers have a long history of promoting comprehensive health services, but using them to deliver infectious disease prevention services in the United States has not been well studied recently. When to use community health workers and the skills required vary depending on the environment, culture, and context in which they are used. In some environments, where healthcare services may be difficult to access, community health workers may be involved in various stages of infectious disease prevention: general health promotion, specific prophylaxis, early diagnosis and treatment, limiting disability, and rehabilitation. In this context, community health workers may be junior level health officers or volunteer healthcare workers who receive appropriate training for functioning at the required stage.

In other environments, community health workers function as *promotoras* or "health promoters." In this context, promotoras typically emerge from naturally occurring networks and focus on fostering behavior change through role modeling, group activities, skill building, and goal setting. Promotoras work in small groups and build strong collaborations with schools, churches, clinics, and local health departments. Through these activities, promotoras facilitate links between patients and the healthcare system and minimize dropouts from required treatment regimens. Promotoras and community healthcare workers are trusted by the communities they serve and provide cost-effective services to persons whose infectious diseases might otherwise go untreated.

